# Simultaneous Measurement of Volumetric Flowrates of Gas–Liquid Bubbly Flow Using a Turbine Flowmeter

**DOI:** 10.3390/s23094270

**Published:** 2023-04-25

**Authors:** Tomomi Uchiyama, Shogo Miyamoto, Kosuke Horie, Kotaro Takamure

**Affiliations:** 1 Institute of Materials and Systems for Sustainability, Nagoya University, Furo-cho, Chikusa-ku, Nagoya 464-8601, Japan; 2 Graduate School of Informatics, Nagoya University, Furo-cho, Chikusa-ku, Nagoya 464-8601, Japan

**Keywords:** gas–liquid two-phase flow, flowrate measurement, turbine flowmeter, rotor rotational speed, pressure loss

## Abstract

The flowrate measurement of the gas–liquid two-phase flow frequently observed in industrial equipment, such as in heat exchangers and reactors, is critical to enable the precise monitoring and operation of the equipment. Furthermore, certain applications, such as oil and natural gas processing plants, require the accurate measurements of the flowrates of each phase simultaneously. This study presents a method that can simultaneously measure the volumetric flowrates of each phase of gas and liquid two-phase mixtures, *Q_g_* and *Q_l_*, respectively, without separating the phases. The method employs a turbine flowmeter and two pressure sensors connected to the pipes upstream and downstream of the turbine flowmeter. By measuring the rotational speed of the rotor and the pressure loss across the flowmeter, the flowrate of the two-phase mixtures *Q_tp_* = (*Q_g_* + *Q_l_*) and the gas volumetric flowrate ratio *β* = (*Q_g_*/*Q_tp_*) are determined. The values of *Q_g_* and *Q_l_* are calculated as *βQ_tp_* and (1 − *β*)*Q_tp_*. This study also investigates the measurement accuracies for air–water two-phase flows at 0.67 × 10^−3^ ≤ *Q_tp_* ≤ 1.67 × 10^−3^ m^3^/s and *β* ≤ 0.1, concluding that the full-scale accuracies of *Q_tp_*, *β*, *Q_g_*, and *Q_l_* are 3.1%, 4.8%, 3.9%, and 3%, respectively. These accuracies either match or improve the accuracies of similar methods reported in the literature, indicating that the proposed method is a viable solution for the determination of phase-specific flowrates in gas–liquid two-phase mixtures.

## 1. Introduction

When mixtures consisting of gas and liquid phases flow, the two phases interact with each other. Such gas–liquid two-phase flows are frequently observed in industrial equipment, such as in heat exchangers and reactors. Therefore, the flowrate measurement of a two-phase flow is critical to enable the precise monitoring and operation of the equipment. Furthermore, certain applications, such as oil and natural gas processing plants, require the accurate measurement of the flowrates of each phase simultaneously. In this context, various methods for the measurement of the flowrate of two-phase flows in circular pipes have been proposed [1,2,3,4,5]. To measure the sum of the flowrates of the gas and liquid phases or the flowrate of two-phase mixtures, some proposed methods combine a flowmeter for single-phase flows with a device for measuring the gas volume fraction. Examples include orifice flowmeters and void fraction meters [6,7], turbine flowmeters and electrical conductivity meters [8], venturi flowmeters and void fraction meters [7,9], venturi flowmeters and capacitance tomography [10,11], venturi flowmeters and vortex flowmeters [12], and ultrasonic flowmeters and Coriolis flowmeters [13]. There is an urgent need for a method that can measure the flowrates of both the gas and liquid phases simultaneously in detail and in real-time, without separating the phases. Minemura et al. [14] proposed a turbine flowmeter combined with a two-phase flow homogenizer. They demonstrated that, by reading the rotational speed of the rotor and the difference in the pressures upstream and downstream of the device, the gas and liquid volumetric flowrates can be measured with accuracies of 3.2% and 1.5% of the maximum rated flowrates, respectively. Meanwhile, Li et al. [15] extended the application of an orifice flowmeter for water single-phase flow to two-phase flows. In an alternative approach, Wang et al. [16] proposed a method using a neural network to process the signals from a Coriolis flowmeter and a differential pressure meter to determine phase-specific flowrates.

In a previous work [17], a turbine flowmeter was developed with functions for self-power generation and wireless communication via the Internet. The proposed flowmeter, using a propeller-type rotor with four blades, is connected in series to a circular pipe through which single-phase water flows. Rotating with the water flow, the rotor generates electrical power, which is supplied to a microcomputer and a low-power wide-area (LPWA) wireless communication module. The LPWA communication module uploads the rotational speed of the rotor, which is measured by a microcomputer, to a server on the Internet. This self-powered flowmeter lends itself to integration with the Internet of Things (IoT) sensors, which can download the rotor speed data from the server and calculate the flowrate based on a one-to-one relationship between the flowrate and the rotational speed of the rotor.

In a study [17], a maximum power generation efficiency of 6.6% and an output power of 0.245 W were obtained at a flowrate of 0.0012 m^3^/s, and the generated power was confirmed to be sufficient for operating the microcomputer and LPWA wireless communication module. The flowrate measurement accuracy was demonstrated to be 1.2%. In a follow-up study [18], a cone was installed at the front center of the rotor to increase the power generation capability of the system by improving the efficiency of energy conversion from water flow to rotor rotation. The power generation efficiency was demonstrated to be maximized when using a cone with a diameter of 0.375*D*, where *D* is the rotor diameter, resulting in 1.12 times the efficiency of a rotor without a cone.

Based on the aforementioned research, this study proposes a method for the simultaneous measurement of the volumetric flowrates of each phase of gas and liquid two-phase mixtures without separating the phases. The proposed method employs a turbine flowmeter [17] for water single-phase flow. As in [17], the flowmeter has a power generation function; however, as the focus of this study is on the development of the measurement method, the generated power is not utilized for communication. Minemura et al. [14] proposed a similar method for the simultaneous measurement of phase flowrates, also using a turbine flowmeter as described above. However, in their study, a two-phase flow homogenizer with a honeycomb structure was installed upstream of the turbine flowmeter, causing considerable pressure loss. In contrast, to reduce pressure loss across the flowmeter, the flowmeter used in this study does not make use of such a flow homogenizer. The proposed measurement method is applied to air–water two-phase flows, and its accuracy is evaluated against those of existing flowrate measurement methods.

## 2. Turbine Flowmeter and Experimental Procedure

### 2.1. Turbine Flowmeter

In [17], a turbine flowmeter was developed for water single-phase flow, with a power generation function. It was based on the design of a small propeller-type micro-hydraulic turbine developed in a separate study [19]. The current study presents a method for simultaneously measuring the flowrates of each phase of the gas and liquid two-phase mixtures using a turbine flowmeter adapted from the above sources.

**Figure 1:**
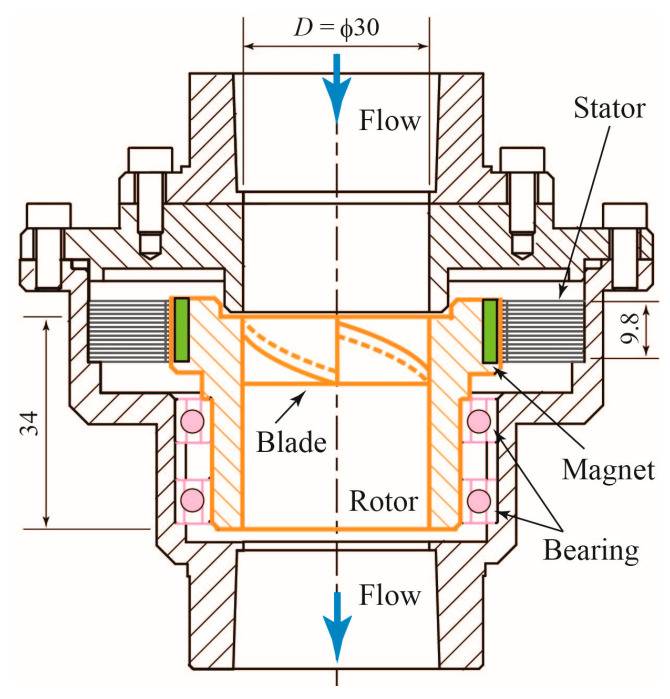
Cross-sectional view of the turbine flowmeter.

A two-dimensional (2D) cross-sectional schematic view of the turbine flowmeter is shown in Figure 1. The flowmeter is connected in series to a circular pipe through which the gas–liquid two-phase mixture flows. The rotor is mounted on the central axis and supported by two bearings. The axial length of the rotor is 34 mm, and the nominal diameter *D* is 30 mm. The rotor rotates around the central axis, under the influence of the two-phase mixture flowing in the axial direction. It is surrounded by a stator core consisting of 28 stacked 0.35 mm thick electromagnetic steel plates. As 14 neodymium magnets are embedded in the outer periphery of the rotor, the flowmeter is also effectively a 3-phase alternator. The flowmeter in [17] was applied to the water single-phase flow measurement and the generated power was used to detect the rotational speed of the rotor and communicate with a server on the Internet. In this study, the generated power is not used for communication; conversely, it is used to measure the number of zero-crossing points in the voltage over a certain time to determine the rotational speed of the rotor.

**Figure 2:**
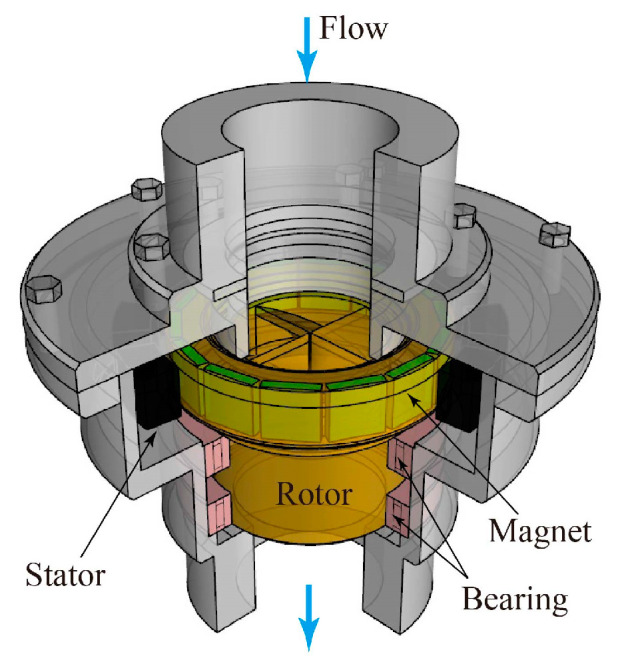
Three-dimensional cross-sectional view of the turbine flowmeter.

**Figure 3:**
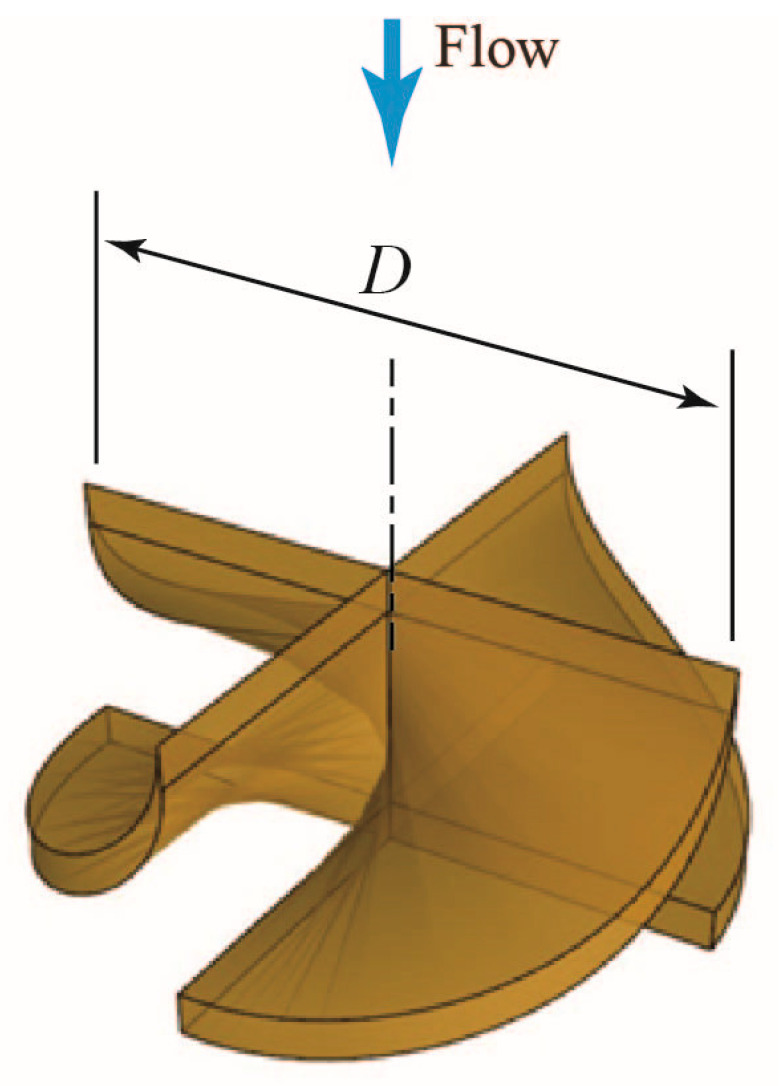
Three-dimensional view of rotor blades.

A three-dimensional (3D) cross-sectional view of the turbine flowmeter is shown in Figure 2, whereas Figure 3 depicts a 3D view of the blade shapes. The rotor has an outer diameter *D* of 53 mm, with 4 blades joined on the central axis of rotation.

**Figure 4:**
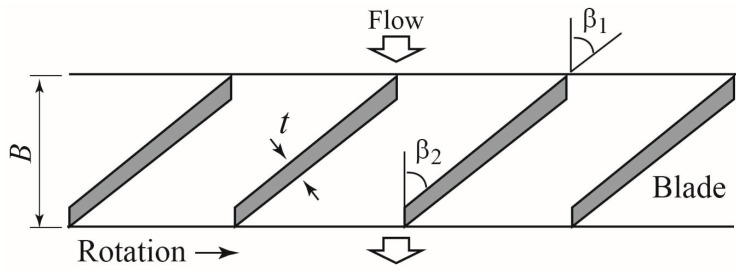
Two-dimensional developmental view of rotor blades at the rotor outer periphery.

Figure 4 depicts a 2D developmental view of the rotor blades at the outer periphery (*r*/*(D*/2) = 1), where *r* refers to the radial position of reference and *D* refers to the outer diameter of the rotor. The rotor is a flat-plate cascade with a blade thickness t of 1.6 mm. Each blade has a width *B* of 11 mm. The inlet angle *β*_1_ and outlet angle *β*_2_ of the blades are equal at all radial positions.

**Figure 5:**
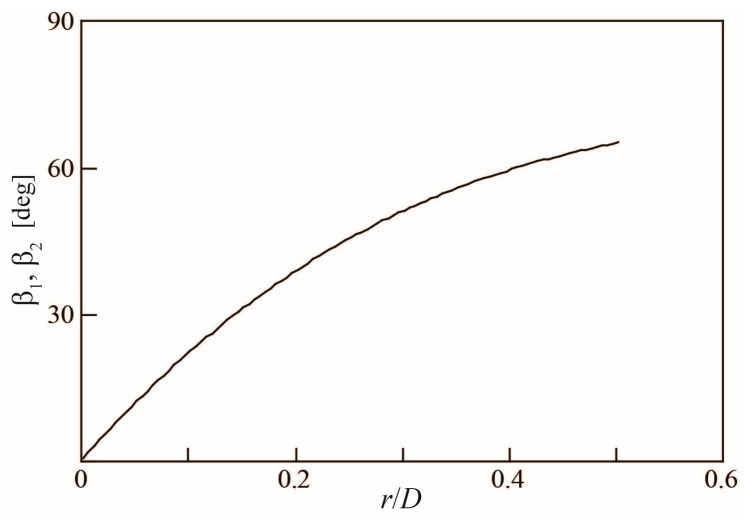
Change in angles *β*_1_ and *β*_2_ of rotor blades with radial position.

The variations in *β*_1_ and *β*_2_ in the radial (*r*) direction are shown in Figure 5. The values are 0 degree at the center (*r*/*D* = 0) and 65 degrees on the outer periphery (*r*/*D* = 0.5).

### 2.2. Experimental Procedure

**Figure 6:**
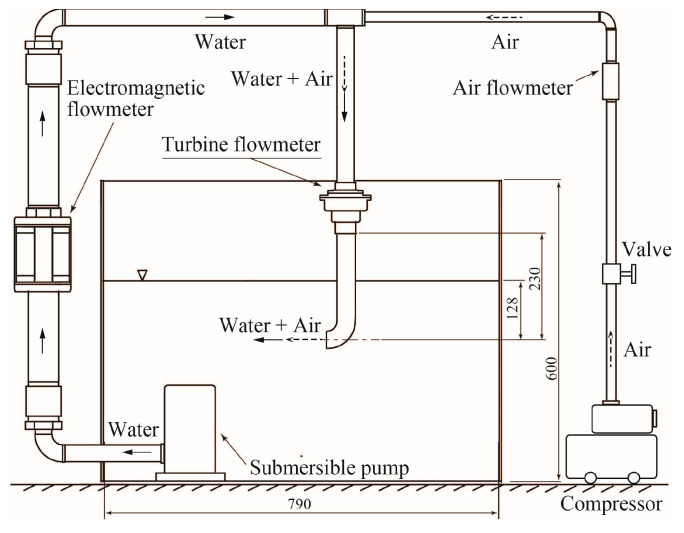
Closed-loop flowrate measurement test rig.

The performance of the turbine flowmeter was investigated using the experimental setup shown in Figure 6. The water was stored in a rectangular tank with a 790 mm width × 790 mm depth × 600 mm height. The water, pumped by a submersible pump, passed through a commercial electromagnetic flowmeter, before flowing through the custom turbine flowmeter and being circulated to a tank. The outlet of the pipe downstream of the turbine flowmeter was located at a depth of 128 mm below the water surface. The water volumetric flowrate *Q_l_*, measured by the electromagnetic flowmeter, was controlled by the inverter drive of the pump. The air from a compressor passed through a flow-control valve and a commercial thermal flowmeter, before being combined with the pumped water flow through a nozzle upstream of the turbine flowmeter. In this way, a two-phase flow was achieved. The air volumetric flowrate *Q_g_*, measured by the thermal flowmeter, could be set independently of *Q_l_*. The measurement ranges of the commercial electromagnetic and thermal flowmeters were 4.17 × 10^−4^ − 1.67 × 10^−2^ m^3^/s and 33 × 10^−6^ − 1.67 × 10^−4^ m^3^/s, respectively. Both flowmeters had a full-scale accuracy of 1 %. The flowrate of the two-phase mixtures *Q_tp_* was therefore (*Q_g_* + *Q_l_*), and the gas volumetric flowrate ratio *β* was defined as *β* = *Q_g_*/*Q_tp_*. The experiments in this study were conducted at 0.67 × 10^−3^ ≤ *Q_tp_* ≤ 1.67 × 10^−3^ m^3^/s and 0 ≤ *β* ≤ 0.1.

**Figure 7:**
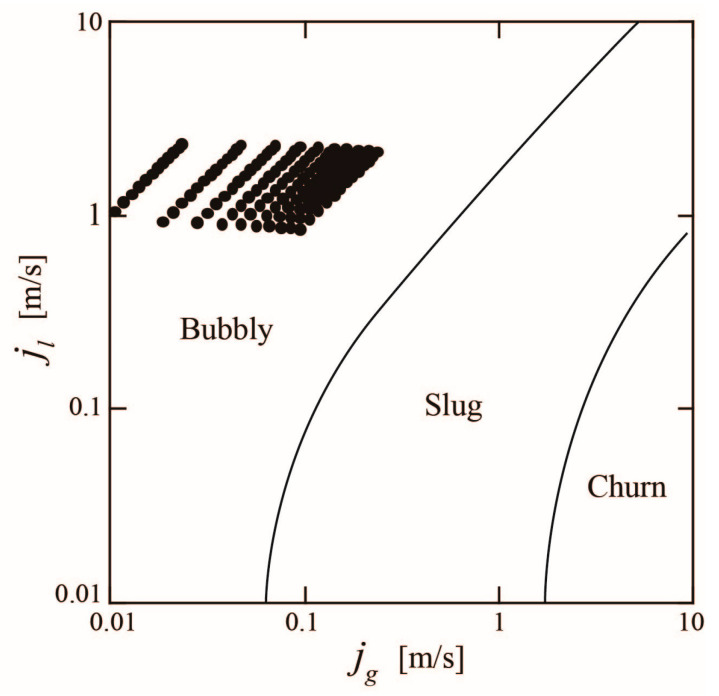
Flow regime map and experimental conditions.

Figure 7 shows the flow regime map [20] for the air–water two-phase flow at ambient temperature and atmospheric pressure. The gas flux *j_g_* and liquid flux *j_l_* are given by *Q_g_*/(*πD*^2^/4) and *Q_l_*/(*πD*^2^/4). The fluxes at which the experiments were conducted are indicated by the circular symbols in Figure 7. The fluxes were in the bubbly flow regime. Bubbly flows occur when small gas bubbles are dispersed within a liquid flow, which is common in industrial equipment. The objective of this study was to develop a turbine flowmeter for bubbly flow, so the values of *Q_tp_* and *β* were set so that bubbly two-phase mixtures flow inside the turbine flowmeter.

**Figure 8:**
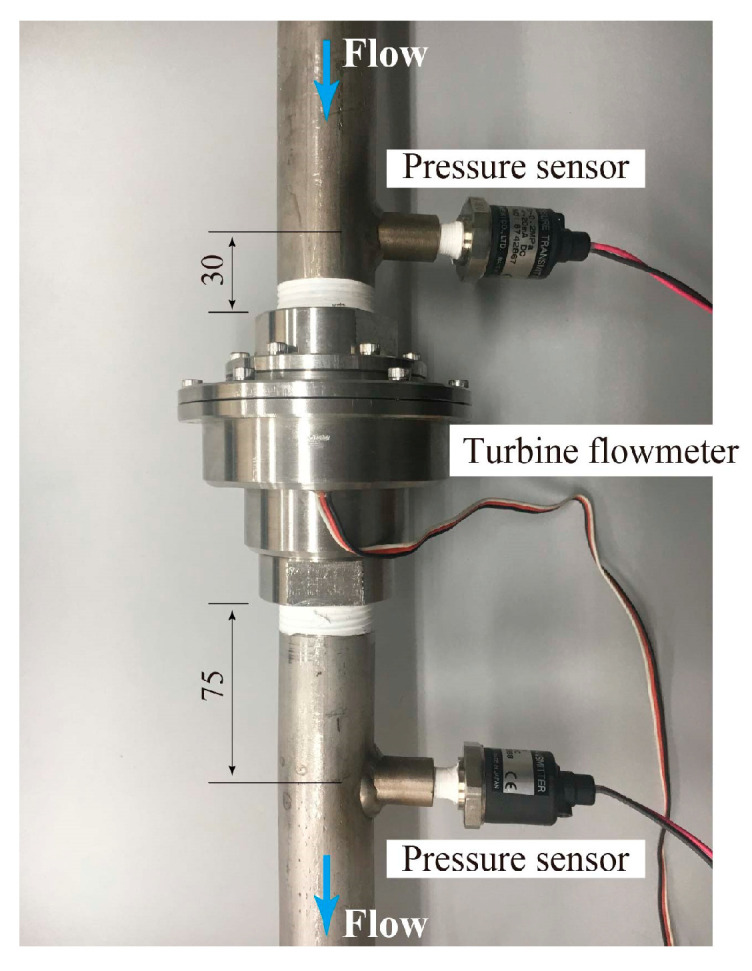
Turbine flowmeter and pressure sensors.

As shown in Figure 8, the semiconductor pressure sensors are connected to the pipes 30 mm upstream and 75 mm downstream of the turbine flowmeter to measure the pressures *p*_1_ and *p*_2_, respectively. The holes in the wall of the pipes connected to the turbine flowmeter were used to measure *p*_1_ and *p*_2_. The holes had a diameter of 2 mm and were carefully punched, avoiding the creation of uneven surfaces on the pipe wall and the alteration of either the flow in the pipes or the performance of the turbine flowmeter. Because the turbine flowmeter is a three-phase AC generator, the rotor rotational speed *N* is detected by reading the number of zero-crossing points in the generator voltage over a fixed period. In [17], the one-to-one relationship between *N* and the flowrate *Q_l_* for the water single-phase flow is demonstrated, thus enabling the identification of *Q_l_* from the measured *N* value. This study proposes a similar method to simultaneously identify the gas and liquid flowrates *Q_g_* and *Q_l_*, respectively, by measuring *N* and the pressure difference (*p*_1_ − *p*_2_). This study measured the rotational speed of the rotor, pressures, and air and water flowrates over 20 s, repeating the measurements 3 times to calculate their averaged values.

## 3. Rotor Angular Velocity and Pressure Loss

### 3.1. Rotation of Rotor Due to Water Single-Phase Flow

**Figure 9:**
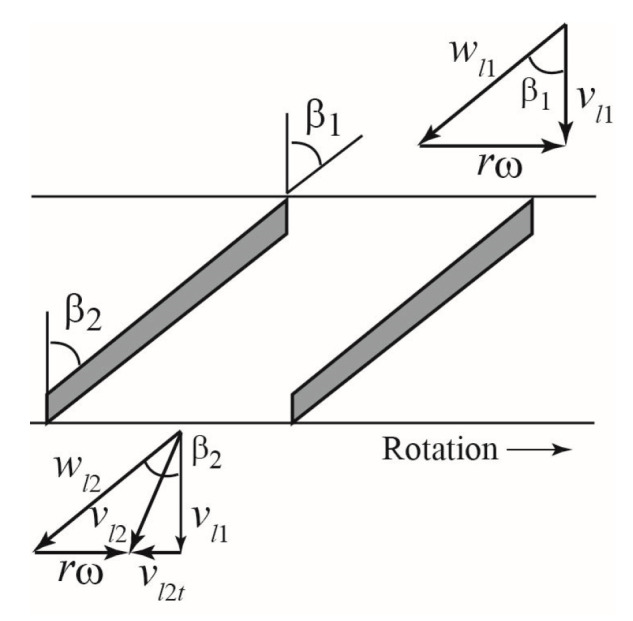
Velocity triangles at rotor inlet and outlet.

Figure 9 shows a cross-section of the flow passage between the two blades in the radial position *r*. First, a turbine flowmeter operated for the water single-phase flow was considered. The rotor was assumed to rotate at a constant angular velocity *ω*. It was also postulated that the water flows along the rotor axis at the rotor inlet and that the water velocity is distributed uniformly at the rotor inlet and outlet. The water velocity relative to the rotor was denoted by *w_l_* and the absolute velocity was denoted by *v_l_*. The velocity triangles at the rotor inlet and outlet are shown in Figure 9, where the subscripts 1 and 2 represent the inlet and outlet, respectively. Consider a blade element with a small radius Δ*r* at a radial position *r*, when the water density is *ρ_l_*, the torque Δ*T_l_* produced on the blade element by the water flow is given using the following equation,

(1) $$\Delta T_{l}=\rho_{l}rv_{l2t}\Delta Q_{l},$$

where *v_l_*_2*t*_ denotes the circumferential component of *v_l_* at the rotor outlet and Δ*Q_l_* denotes the flowrate of the water flowing through the ring conduit of the radius Δ*r*. The parameters are calculated using the following equations:

(2) $$v_{l2t}=v_{l1}\tan\beta_{2}-r\omega ,$$

(3) $$\Delta Q_{l}=2\pi r\Delta rv_{l1}.$$

The torque *T_l_* produced on the entire blade can be obtained by integrating Equation (1) from the center of the rotor (*r* = 0) toward the periphery (*r* = 0.5*D*). The blade angle *β*_2_ is represented by the value *β*_2*m*_ at the average rotor radius *r_m_* (*r_m_* = $\sqrt{2}D/4$). The velocity *v_l_*_2*t*_ is represented by the value *v_l_*_2*tm*_ at *r* = *r_m_* and the water flowrate *Q_l_* through the rotor is expressed as 2*π*(*r_m_*)^2^*v_l_*_1_. *T_l_* is given using the following equation,

(4) $$T_{l}=\rho_{l}r_{m}v_{l2tm}Q_{l}=r_{m}^{2}\rho_{l}Q_{l}\left(\frac{\tan\beta_{2m}}{2\pi r_{m}^{3}}Q_{l}-\omega \right),$$

where *T_l_* is equal to the sum of the mechanical friction torque *T_m_* due to the bearings, the fluid resistance torque *T_f_*, and the cogging torque *T_c_* due to the magnetic attractive forces [21,22] that prevent the rotor from rotating.

(5) $$T_{l}=T_{m}+T_{f}+T_{c}.$$

By substituting Equation (5) into Equation (4) and solving the resulting equation for *ω*, the following equation is obtained:

(6) $$\omega =\frac{\tan\beta_{2m}}{2\pi r_{m}^{3}}Q_{l}-\frac{T_{m}+T_{f}+T_{c}}{r_{m}^{2}\rho_{l}Q_{l}}.$$

### 3.2. Rotation of Rotor Due to Gas–Liquid Two-Phase Flow

After derivation of the equations for the water single-phase flow, the flow of gas–liquid two-phase mixtures through the rotor was considered. The rotor was assumed to rotate at a constant angular velocity *ω*, as with the water single-phase flow. It was postulated that the gas and liquid phase flow along the rotor axis at the rotor inlet and that they both have uniform velocities in the circumferential direction at the rotor inlet and outlet. The gas velocity relative to the rotor and absolute velocity were defined as *w_g_* and *v_g_*, respectively. Assuming that the density of the gas phase is *ρ_g_*, the torque Δ*T_tp_* produced on the blade element Δ*r* by two-phase flow can be expressed as follows,

(7) $$\Delta T_{tp}=\rho_{g}rv_{g2t}\Delta Q_{g}+\rho_{l}rv_{l2t}\Delta Q_{l},$$

where *v_g_*_2*t*_ denotes the circumferential component of *v_g_* at the rotor outlet, and Δ*Q_g_* and Δ*Q_l_* represent the flowrates of the gas and liquid phases, respectively, through a ring conduit with the radius Δ*r*. The above parameters can be calculated using the following equations,

(8) $$v_{g2t}=v_{g1}\tan\beta_{2}-r\omega ,$$

(9) $$\Delta Q_{g}=2\pi r\Delta r\alpha v_{g1},$$

(10) $$\Delta Q_{l}=2\pi r\Delta r\left(1-\alpha \right)v_{l1},$$

where *α* denotes the gas volume fraction.

When using the gas and liquid flowrates through the rotor, *Q_g_* ($=2\pi r_{m}^{2}\alpha v_{g1}$) and *Q_l_* ($=2\pi r_{m}^{2}\left(1-\alpha \right)v_{l1}$), respectively, the torque *T_tp_* produced on the entire blade is given using the following equation:

(11) $$\begin{array}{c} T_{tp}=\rho_{g}r_{m}v_{g2tm}Q_{g}+\rho_{l}r_{m}v_{l2tm}Q_{l}\\ =\tan\beta_{2m}\left(\rho_{g}r_{m}Q_{g}v_{g1}+\rho_{l}r_{m}Q_{l}v_{l1}\right)-r_{m}^{2}\omega \left(\rho_{g}Q_{g}+\rho_{l}Q_{l}\right). \end{array}$$

When the flowrate of a two-phase mixture is expressed as *Q_tp_* = (*Q_g_* + *Q_l_*), Equation (11) can be rewritten as follows:

(12) $$T_{tp}=r_{m}^{2}\left(\rho_{g}Q_{g}+\rho_{l}Q_{l}\right)\left[2\pi r_{m}\tan\beta_{2m}\frac{\rho_{g}\alpha v_{g1}^{2}+\rho_{l}\left(1-\alpha \right)v_{l1}^{2}}{\rho_{g}Q_{g}+\rho_{l}Q_{l}}-\omega \right].$$

Solving Equation (12) for *ω* and assuming that *T_tp_* is equal to the sum of the mechanical friction torque *T_m_*, fluid drag torque *T_f_*, and cogging torque *T_c_*, as in Equation (5) for the case of water single-phase flow, the following equation is obtained:

(13) $$\omega =\frac{\tan\beta_{2m}}{2\pi r_{m}^{3}}\frac{as^{2}+1}{\left(as+1\right)\left[1-\alpha \left(1-s\right)\right]}Q_{tp}-\frac{T_{m}+T_{f}+T_{c}}{r_{m}^{2}\left(\rho_{g}Q_{g}+\rho_{l}Q_{l}\right)}.$$

Here, *a* and *s* are the two-phase flow coefficients and velocity ratio, respectively, as defined by the following equations:

(14) $$a=\frac{\alpha \rho_{g}}{\left(1-\alpha \right)\rho_{l}},$$

(15) $$s=\frac{v_{g1}}{v_{l1}}.$$

### 3.3. Pressure Loss Multiplier

To predict the pressure loss of gas–liquid two-phase flows, Δ*p_tp_*, the pressure loss multiplier $\phi_{l}^{2}$ is widely used. It is defined by the following equation [23],

(16) $$\phi_{l}^{2}=\frac{\Delta p_{tp}}{\Delta p_{l0}},$$

where Δ*p_l_*_0_ denotes the pressure loss when only the liquid phase flows with the same flowrate.

Akagawa [24] has shown that $\phi_{l}^{2}$ of air–water two-phase flows in the horizontal, inclined, and vertical pipes at room temperature under atmospheric pressure can be expressed through the following equation using the air volume fraction *α*,

(17) $$\phi_{l}^{2}={\left(1-\alpha \right)}^{-z},$$

where *z* is a constant and 1.4 ≤ *z* ≤ 1.9. The pressure loss under atmospheric pressure, derived by Lockhart–Martinelli [23], can be approximated using *z* = 1.975 [24].

As mentioned earlier, this study used pressure sensors connected to the pipes 30 mm upstream and 75 mm downstream of the turbine flowmeter, as shown in Figure 8, to measure the pressures *p*_1_ and *p*_2_, respectively. The pressure difference (*p*_1_ − *p*_2_) corresponds to the pressure loss Δ*p_tp_* across the turbine flowmeter.

## 4. Experimental Results and Discussion

### 4.1. Rotor Angular Velocity and Pressure Loss for Water Single-Phase Flow

**Figure 10:**
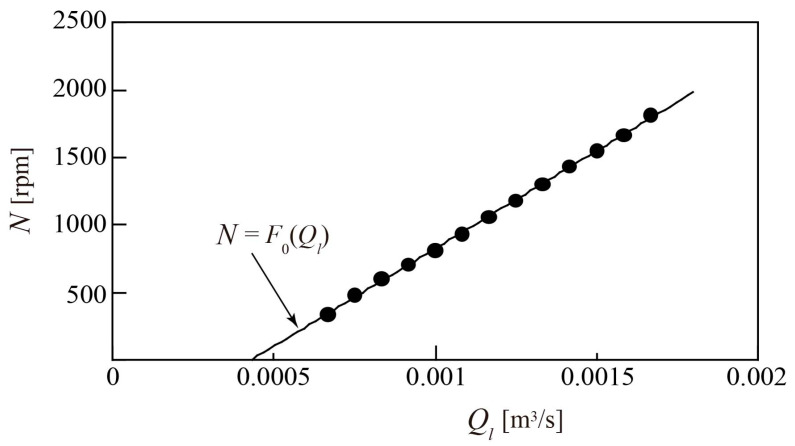
Relationship between water flowrate *Q_l_* and rotor rotational speed *N* for water single-phase flow.

The first experiments in this study were carried out for the water single-phase flow. Figure 10 shows the relationship between the water flowrate *Q_l_*, measured using an electromagnetic flowmeter, and the rotor rotational speed *N*. The value of *N* can be calculated based on the angular velocity ω on the left side of Equation (6), and is given by 60*ω*/2*π*, expressed in rpm. In the range of *Q_l_* ≥ 0.67 × 10^−3^ m^3^/s, the water flow causes the rotor to rotate, and the relation *N* > 0 is obtained. In this flowrate range, *N* can be approximated by a linear function *F*_0_ of *Q_l_*. The function is plotted as a solid line in Figure 10, where *N* increases linearly with increasing *Q_l_*. The rate of change in *N* owing to *Q_l_* depends on tan*β*_2*m*_/2π(*r_m_*)^3^, which represents the rotor geometry, as determined from the first term on the right side of Equation (6). It also depends on the effect of the torques in the second term on the right side of Equation (6); however, this is sufficiently small compared to the first term to make it negligible. Therefore, *Q_l_* can be identified by substituting the measured *N* value into the function *F*_0_. It should be noted that the function *F*_0_ does not pass through its origin. It is therefore considered that, when *Q_l_* ≤ 0.43 × 10^−3^ m^3^/s, *N* is zero, and the rotor does not rotate. This is due to the effect of the sum of torques in Equation (6) becoming sufficiently large to prevent the rotor from rotating. The cogging torque caused by the magnetic attractive forces amplifies this.

**Figure 11:**
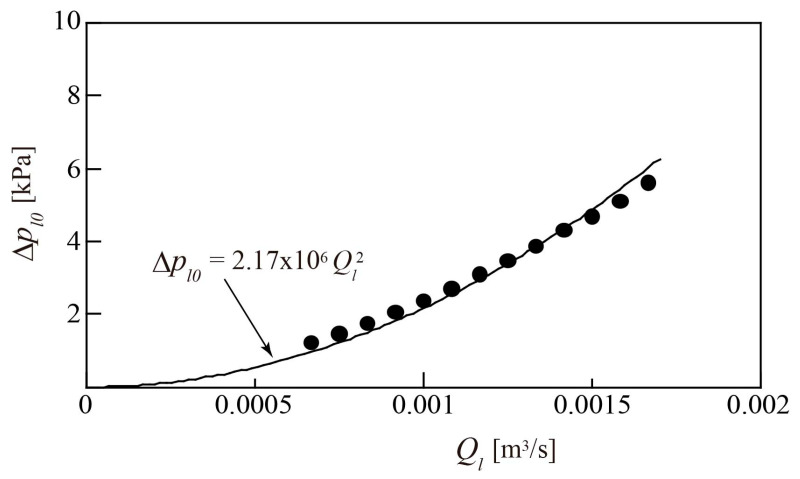
Relationship between water flowrate *Q_l_* and pressure loss Δ*p_l_*_0_ for water single-phase flow.

The pressure difference Δ*p_l_*_0_ across turbine flowmeter is plotted against *Q_l_* in Figure 11, where the static pressures upstream and downstream of the turbine flowmeter are taken into account. The value of Δ*p_l_*_0_ increases with *Q_l_*. This can be approximated by a quadratic function of *Q_l_*, as indicated by the solid line in Figure 11.

### 4.2. Rotor Angular Velocity and Pressure Loss for Gas–Liquid Two-Phase Flow

**Figure 12:**
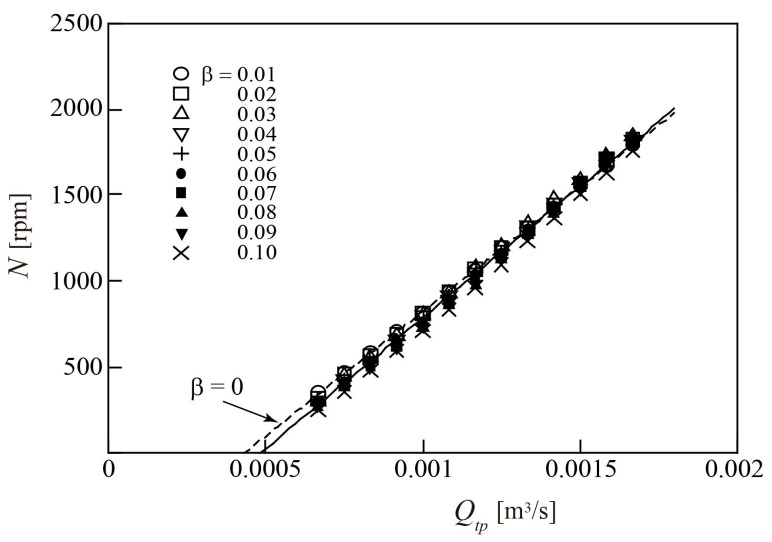
Relationship between two-phase flowrate *Q_tp_* and rotor rotational speed *N*.

The gas flowrate *Q_g_* and liquid flowrate *Q_l_* of the two-phase mixtures were measured using a thermal flowmeter and electromagnetic flowmeter, respectively. Figure 12 shows the relationship between the rotor rotational speed *N* and the total flowrate of the mixtures *Q_tp_* = (*Q_g_* + *Q_l_*). The results are plotted for 0.01 ≤ *β* ≤ 0.1, where *β* is the gas volumetric flowrate ratio (*Q_g_*/*Q_tp_*). When *Q_tp_* ≥ 0.67 × 10^−3^ m^3^/s, the rotor rotates and *N* > 0, irrespective of β. The relationship between *N* and *Q_tp_* is nearly independent of *β*, except for the case of *β* = 0.1, and *N* increases linearly with *Q_tp_*. For all measurements, *N* can be approximated by a linear function of *Q_tp_*, plotted as a solid line in Figure 12. This approximation almost coincides with the relationship derived for the water single-phase flow (*β* = 0), indicated by a dashed line in Figure 12; the coincidence improves for larger *Q_tp_*.

The two-phase flow parameter a and velocity ratio s, defined by Equations (14) and (15), respectively, are included in the first term on the right side of Equation (13). In a turbine flowmeter, the shearing effect of the rotating blades causes the gas phase to form small bubbles. Therefore, in such cases, the gas velocity is almost equal to the liquid velocity; thus, *s* ≃ 1 and *α* ≃ *β*. Furthermore, *a* « 1 is satisfied because *β* is relatively low. In this case, the first term on the right side of Equation (13) is $\left(\tan\beta_{2m}/2\pi r_{m}^{3}\right)Q_{tp}$ and the rate of change of *N* with respect to *Q_tp_* (two-phase flow) coincides with that of *N* with respect to *Q_l_* for the water single-phase flow, as derived from Equation (6). When *Q_tp_* is high, the second term on the right side of Equation (13) is smaller than the first term, as in the case of the water single-phase flow. Thus, the relationship between *Q_tp_* and *N* at *Q_tp_* ≥ 0.001 m^3^/s and *β* ≤ 0.1 for the gas–liquid two-phase flow can be understood as almost identical to that for the water single-phase flow (*β* = 0).

**Figure 13:**
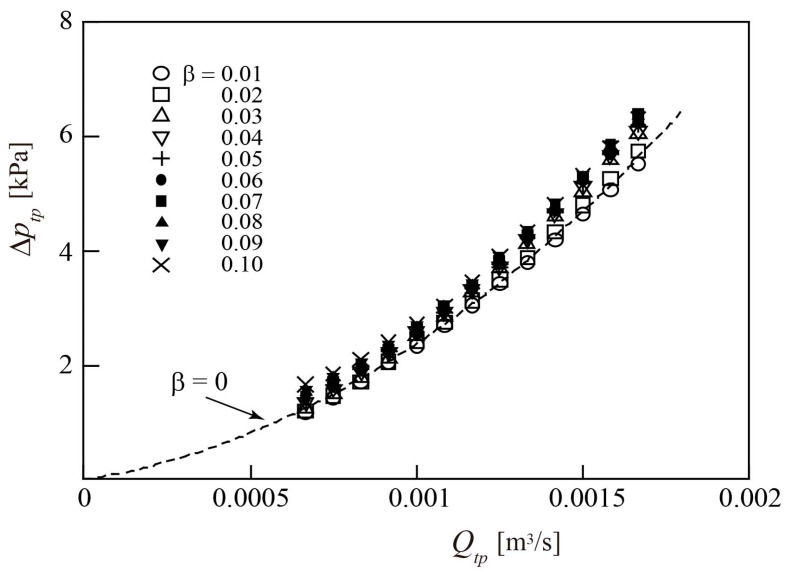
Relationship between two-phase flowrate *Q_tp_* and rotor pressure loss Δ*p_tp_*.

Figure 13 shows the relationship between the pressure difference Δ*p_tp_* = (*p*_1_ − *p*_2_) across the turbine flowmeter and *Q_tp_* for 0.01 ≤ *β* ≤ 0.1. The corresponding relationship for the water single-phase flow (*β* = 0) is plotted using a dashed line. Irrespective of the β value, the pressure loss Δ*p_tp_* increases with increasing *Q_tp_*; however, the increase is more pronounced for higher values of *β*.

**Figure 14:**
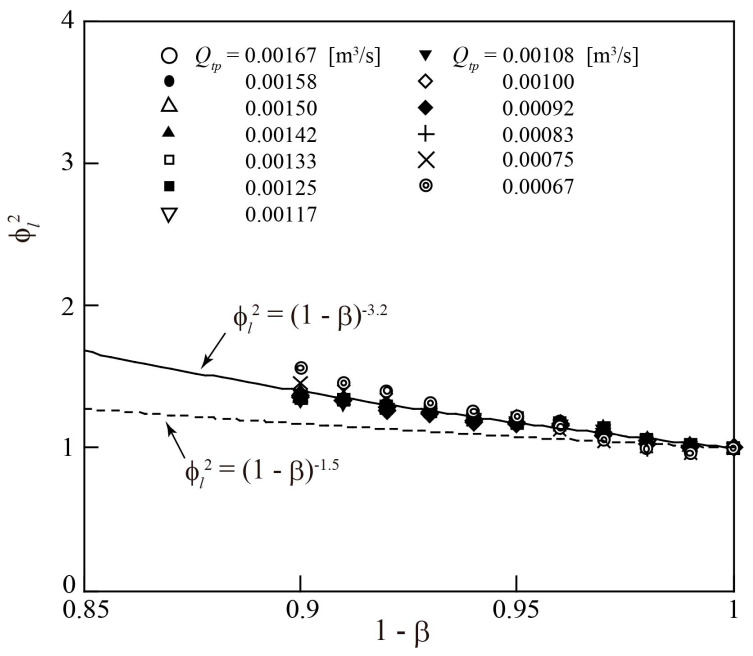
Change in pressure loss multiplier $\phi_{l}^{2}$ with gas flowrate ratio *β*.

Substituting the Δ*p_l_*_0_ and Δ*p_tp_* shown in Figure 11 and Figure 13, respectively, into Equation (16) yields the pressure loss multiplier $\phi_{l}^{2}$. Since α ≃ β in the turbine flowmeter, as explained above, *α* can be replaced by *β* in Equation (17). Figure 14 illustrates the relationship between $\phi_{l}^{2}$ and (1 − *β*) for 0.67 × 10^−3^ ≤ *Q_tp_* ≤ 1.67 × 10^−3^ m^3^/s. The pressure loss multiplier $\phi_{l}^{2}$ increases with increasing *β*, except when *Q_tp_* = 0.67 × 10^−3^ m^3^/s, where *Q_tp_* hardly affects $\phi_{l}^{2}$. These results are consistent with the relationship predicted when *z* is set to 3.2 in Equation (17). Note that $\phi_{l}^{2}$ is slightly larger than the value of (1 − *β*)^−1.5^ obtained by Akagawa [24] for vertical circular pipes; this is attributed to the large pressure loss caused by the rotor in the turbine flowmeter.

### 4.3. Measurement Method for Gas and Liquid Flowrates

As shown in Figure 12, the rotor rotational speed *N* is only slightly affected by the gas volumetric flowrate ratio *β*. It has a one-to-one relationship with the flowrate of two-phase mixtures *Q_tp_* as follows,

(18) $$Q_{tp}=F_{1}\left(N\right),$$

where *F*_1_ is the approximated straight-line function indicated by the solid line in Figure 12. Thus, if *F*_1_ is derived in advance, and the measured *N* value is substituted into *F*_1_, the value of *Q_tp_* can be determined.

As shown in Figure 13, the pressure loss Δ*p_tp_* increases consistently with increasing *Q_tp_*, with larger increments corresponding to higher *β* values. Accordingly, *β* can be expressed as a function *F*_2_ of *Q_tp_* and Δ*p_tp_*, as follows:

(19) $$\beta =F_{2}\left(Q_{tp},\Delta p_{tp}\right).$$

Combining Equations (18) and (19) gives the following equation:

(20) $$\beta =F_{2}\left(F_{1}\left(N\right),\Delta p_{tp}\right).$$

**Figure 15:**
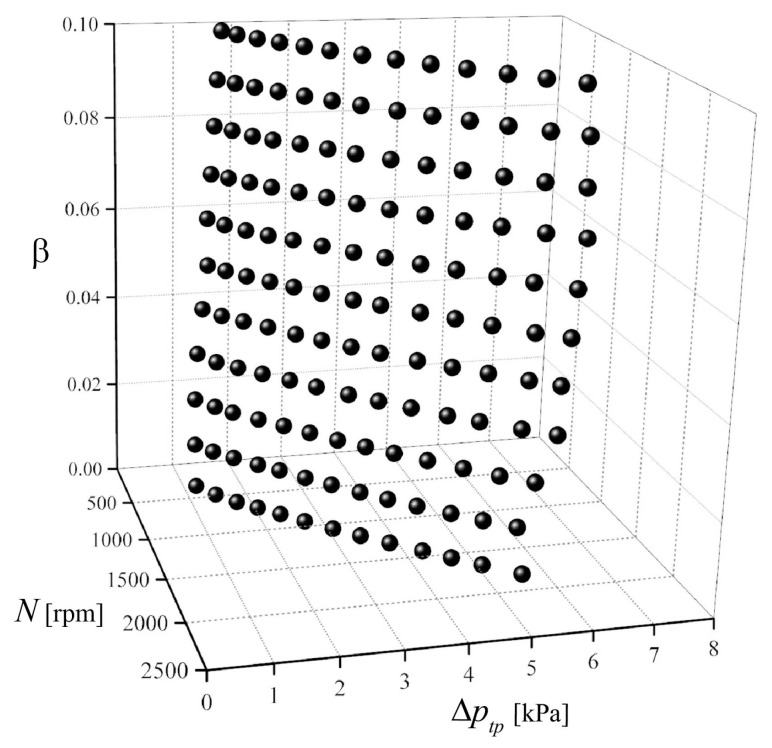
Relationship between *N*, Δ*p_tp_*, and *β*.

Figure 15 shows the relationship between *N*, Δ*p_tp_*, and *β*, used to derive the function *F*_2_. It is thus confirmed that *β* is uniquely determined from the *N* and Δ*p_tp_* values. Accordingly, the value of *β* can be identified by measuring the values of *N* and Δ*p_tp_* and using Equation (20) or Figure 15.

Using *Q_tp_* and *β*, obtained using the above method, the gas and liquid flowrates, *Q_g_* and *Q_l_*, respectively, are derived as *βQ_tp_* and (1 − *β*)*Q_tp_*, respectively.

### 4.4. Flowrate Measurement Accuracy

The accuracy of the measurement method devised above was investigated. The gas flowrate measured by the thermal flowmeter and the liquid flowrate measured by the electromagnetic flowmeter are denoted as *Q_g_*_0_ and *Q_l_*_0_, respectively. The flowrate of the two-phase mixtures, (*Q_g_*_0_ + *Q_l_*_0_), is denoted as *Q_tp_*_0_, and the gas volumetric flowrate ratio *Q_g_*_0_/*Q_tp_*_0_ is defined as *β*_0_.

**Figure 16:**
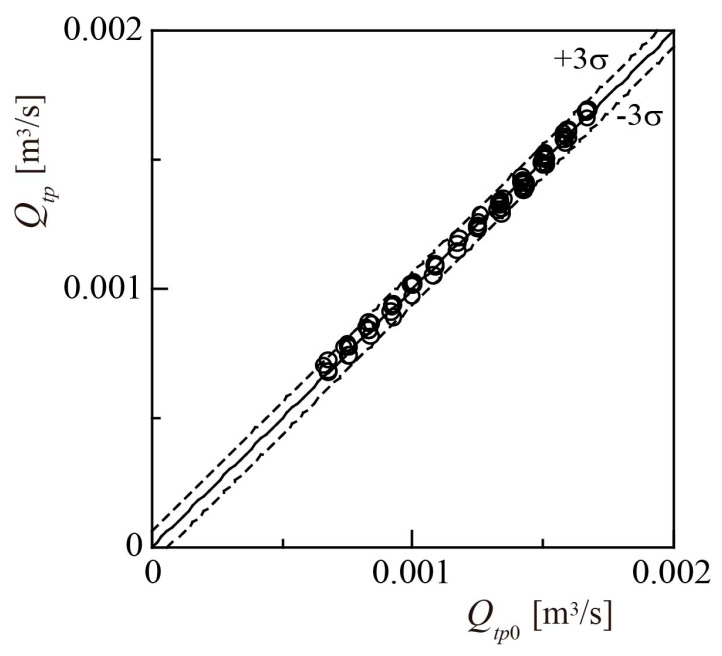
Accuracy of measured two-phase flowrate *Q_tp_*.

The rotor rotational speed *N* was measured and substituted into Equation (18) to obtain the flowrate of the two-phase mixture *Q_tp_*. Figure 16 illustrates the relationship between *Q_tp_* and *Q_tp_*_0_. The standard deviation σ of the difference (*Q_tp_* − *Q_tp_*_0_) was calculated for all measurements, and the values of ±3σ were indicated by dashed lines. The full-scale accuracy, or the ratio of 3σ to the maximum of *Q_tp_*_0_, was 3.1%. The lead-scale accuracy, or the ratio of the maximum value of 3σ to *Q_tp_*_0_, was 5.2%. The full-scale and lead-scale accuracies of the turbine flowmeter of Minemura et al. [14] were 3.1% and 5.1%, respectively, which are almost identical to the accuracies obtained in the present study. However, as mentioned earlier, Minemura et al. [14] installed a two-phase flow homogenizer with a honeycomb structure upstream of turbine flowmeter, resulting in a large pressure loss. In contrast, the present method does not employ such a homogenizer. Zheng et al. [8] proposed a method that combines a turbine flowmeter and electrical conductivity meter, reporting a lead-scale accuracy of 7.9%. Therefore, compared to existing methods, the method developed in this study can measure *Q_tp_* with a high accuracy, and without excessive pressure loss.

**Figure 17:**
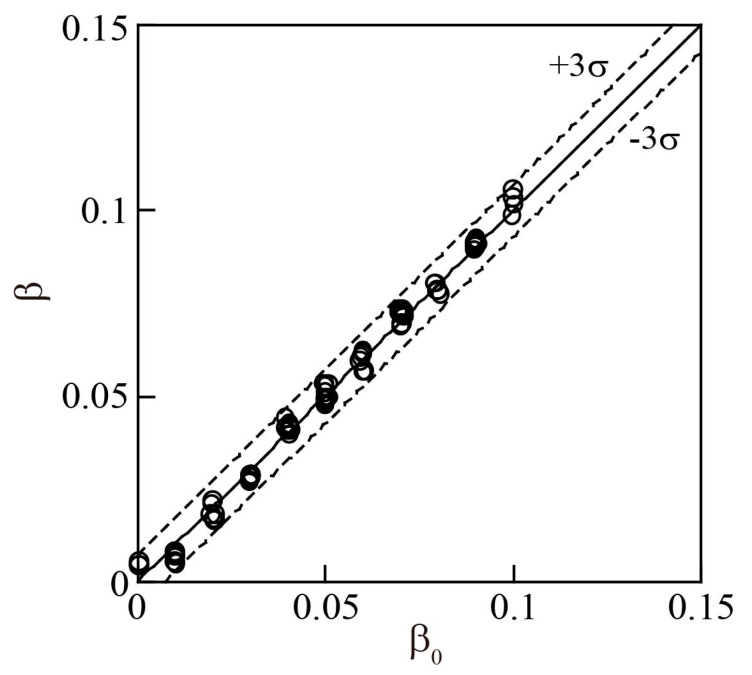
Accuracy of measured gas flowrate ratio *β*.

To calculate *β*, *N* and Δ*p_tp_* were measured and substituted into Equation (20). Figure 17 illustrates the relationship between *β* and *β*_0_. The standard deviation σ of the difference (*β* − *β*_0_) was calculated for all measurements, and the values of ±3σ were indicated by the dashed lines in Figure 17. A value of 3σ had a full-scale accuracy of 4.8%. The *β*_0_ accuracy of Minemura et al. [14] was unclear.

**Figure 18:**
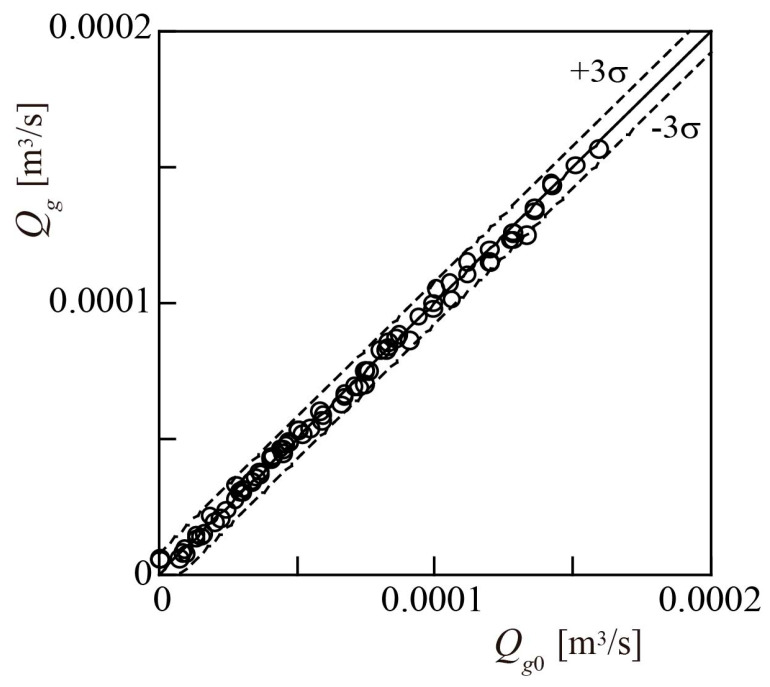
Accuracy of measured gas volumetric flowrate *Q_g_*.

The gas flowrate *Q_g_* = (*βQ_tp_*) was calculated using *Q_tp_* and *β* was obtained using the above method. The results are shown in Figure 18, alongside *Q_g_*_0_. The full-scale accuracy was 3.9%, which is near that of the turbine flowmeter proposed by Minemura et al. [14], which is 3.2%.

**Figure 19:**
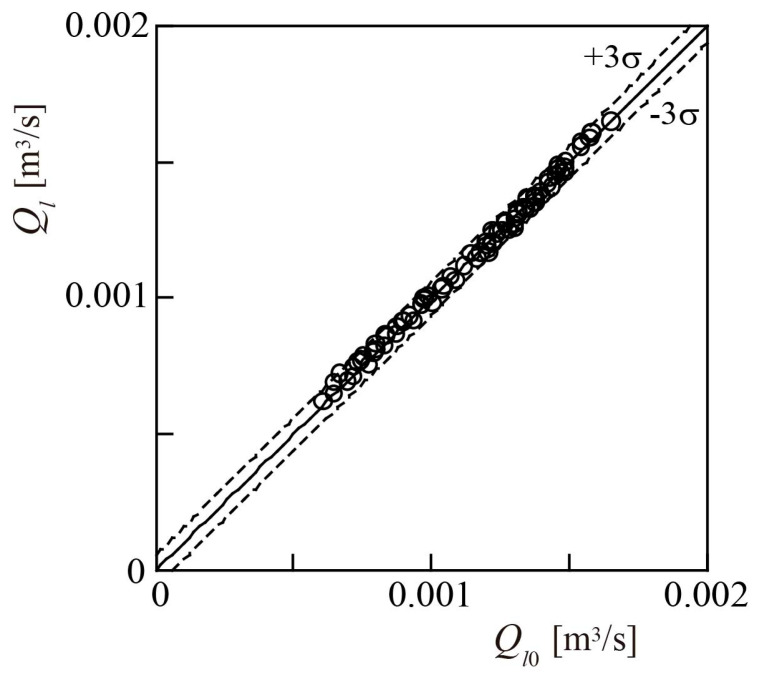
Accuracy of measured liquid volumetric flowrate *Q_l_*.

Figure 19 shows the relationship between the liquid flowrates *Q_l_* [=(1 − *β*)*Q_tp_*] and *Q_l_*_0_. The full- and lead-scale accuracies were 3% and 5%, respectively. These accuracies are almost comparable to those of Minemura et al. [14], at 1.5 % and 4.9%, respectively, but represent a significant improvement on the lead-scale accuracy of 15% reported for the orifice flowmeter proposed by Li et al. [15].

## 5. Conclusions

This study presents a method for the simultaneous measurement of the volumetric flowrate for each phase of gas and liquid two-phase mixtures in a circular pipe, *Q*_g_ and *Q_l_*, respectively, without separating the phases. This method employed a turbine flowmeter and two pressure sensors connected to pipes upstream and downstream of the turbine flowmeter. To investigate the measurement accuracy, the experiments were conducted for air–water two-phase flows. The flowrate of the two-phase mixtures *Q_tp_* = (*Q_g_* + *Q_l_*) was 0.67 × 10^−3^ ≤ *Q_tp_* ≤ 1.67 × 10^−3^ m^3^/s, and the gas volumetric flowrate ratio *β* = (*Q_g_*/*Q_tp_*) was less than 0.1. The following conclusions were drawn:

1. The rotor rotational speed *N* increases linearly as the flowrate *Q_tp_* increases. The relationship between *N* and *Q_tp_* has little dependance on *β*, except at *β* = 0.1. Therefore, if *Q_tp_* can be expressed as a function of *N* in advance, *Q_tp_* can be identified by measuring *N*.
2. The pressure difference across the turbine flowmeter Δ*p_tp_*, which corresponds to the pressure loss across the turbine flowmeter, increases with *Q_tp_*, with larger increments for higher values of *β*. Therefore, since *Q_tp_* can be represented as a function of *N*, *β* can be uniquely identified by measuring *N* and Δ*p_tp_*. Thereafter, the gas flowrate *Q_g_* = (*βQ_tp_*) and liquid flowrate *Q_l_* [=(1 − *β*) *Q_tp_*] values can be derived from the obtained *Q_tp_* and *β* values.
3. The full-scale accuracies of *Q_tp_*, *β*, *Q_g_*, and *Q_l_* of the present method were found to be 3.1%, 4.8%, 3.9%, and 3%, respectively, which are similar to or better than the values reported in the literature.

## Data Availability

The data supporting the results presented in this work are available upon request from the authors.
